# Efficacy of pulse oximetry for early diagnosis of pulmonary embolism after total knee arthroplasty

**DOI:** 10.1186/s43019-023-00207-0

**Published:** 2024-01-21

**Authors:** Ju-Hyung Yoo, Sang-Hoon Park, Hyun-Cheol Oh, Joong-Won Ha, Han-Kook Yoon

**Affiliations:** 1 Department of Orthopedic Surgery, National Health Insurance Service Ilsan Hospital, Ilsan-ro 100, Ilsandong-gu, Goyang, 10444 South Korea; 2 Department of Orthopedic Surgery, Seran Hospital, Seoul, Korea

**Keywords:** Total knee arthroplasty, Pulmonary embolism, Oxygen saturation, Pulse oximeter

## Abstract

**Introduction:**

Early diagnosis and aggressive treatment of pulmonary embolism (PE) are crucial for preventing severe complications after total knee arthroplasty (TKA). This study aimed to examine the efficacy of measuring oxygen saturation (SpO_2_) using a pulse oximeter for early diagnosis of PE after total knee arthroplasty (TKA).

**Materials and methods:**

We consecutively examined 1645 patients who underwent TKA between January 2015 and November 2019. Postoperative SpO_2_ was measured with a pulse oximeter, which was stopped if SpO_2_ was maintained at ≥ 95% until postoperative day 2 (POD2). To diagnose PE, computed tomographic pulmonary angiography (CTPA) was performed for specific indications, including persistently low SpO_2_ < 95% (group 1), sudden decrease in SpO_2_ (group 2), and decrease in SpO_2_ after POD3 with presenting symptoms (group 3). Also, we divided the patients into unilateral, simultaneous and sequential TKA groups and compared the results with specific statistical techniques.

**Results:**

Of the 1645 patients who underwent TKA, there were 20 patients with PE (1.2%), and symptomatic PE was observed in only 4 patients (0.24%). CTPA was performed in 58 (3.5%) patients, of whom 20 were diagnosed with PE. In groups 1 (*n* = 34), 2 (*n* = 21), and 3 (*n* = 3), CTPA was performed 2.4, 2.6, and 8.3 days after TKA, respectively, and 12, 8, and 0 patients were diagnosed with PE, respectively. Of the 782, 416, and 447 unilateral, simultaneous, and sequential (done in same admission with interval 1 or 2 weeks) patients with TKA, 38, 18, and 2 received CTPA, and 13, 6, and 1 were diagnosed with PE, respectively. All patients diagnosed with PE have persistently low SpO_2_ < 95% (group 1), or sudden decrease in SpO2 (group 2) until POD2. Of the patients diagnosed with PE, SpO_2_ decreased without the presentation of symptoms in 16 patients (11 and 5 from groups 1 and 2, respectively) and with the presentation of symptoms, such as mild dyspnea and chest discomfort, in 4 patients (1 and 3 from groups 1 and 2, respectively).

**Conclusions:**

Measuring SpO_2_ using a pulse oximeter until POD2 was an effective method for early diagnosis of PE after TKA. No case of morbidity or mortality was observed after early diagnosis with early stage CTPA and management of PE. We recommend measuring SpO_2_ with a pulse oximeter for early diagnosing of PE in TKA.

## Background

Postoperative venous thromboembolism (VTE), including pulmonary embolism (PE) and deep vein thrombosis (DVT), is one of the most severe complications after total knee arthroplasty (TKA) [1]. In patients who received TKA without thromboprophylaxis, the incidence of PE is reported to be approximately 1.5–10% [2, 3]. Symptomatic PE occurred in 0.41% of 222,684 TKA cases [4]. Moreover, according to a meta-analysis of 18 studies[5], the proportion of patients with symptomatic PE was 0.37% (0.24–0.52%); the proportion was not reduced despite prophylactic anticoagulative measures due to genetic factors. The fatality rate due to PE is estimated to be 7–11% [6–8]. Furthermore, the mortality due to PE after TKA is reported to be 0.07–0.3% when thromboprophylaxis is performed [9, 10]. The inpatient mortality in patients without PE is 0.1%, and with PE is 3.36%; thus, higher mortality is reported in patients with PE [11].

Accordingly, early diagnosis and aggressive treatment of PE are crucial for preventing severe complications regardless of the presenting symptoms [6, 8, 10]. PE is diagnosed using one or more tools: plasma d-dimer levels, computed tomographic pulmonary angiography (CTPA), ventilation–perfusion scintigraphy, pulmonary angiography, magnetic resonance angiography, and echocardiography [3, 5, 7–9]. PE has various presenting features ranging from no symptoms to death; thus, the same diagnostic tools cannot be applied to all patients [6, 10]. When pulmonary artery occlusion occurs due to acute PE, blood flow is redistributed to the non-occluded vessels. It causes ventilation/perfusion (V/Q) mismatch, which leads to inadequate gas exchange, hypoxemia, and hypocapnia [2, 5, 8–10]. Patients can present various clinical symptoms such as dyspnea, tachypnea, chest pain, and so on. In addition, hypoxemia is present in about 74% of patients with acute PE on initial blood gas analysis. Thus, persistent hypoxemia after surgery indicates the possibility of PE.

A non-invasive and convenient method for detecting hypoxemia is a pulse oximeter. In this study, all patients in postoperative care were monitored for hypoxemia using a pulse oximeter. Regardless of the presenting symptoms, patients with a persistent or sudden decrease in oxygen saturation (SpO_2_) underwent CTPA. In the patients diagnosed with PE, the management and prognosis were evaluated. Therefore, this study aimed to examine the efficacy of measuring oxygen saturation (SpO_2_) using a pulse oximeter for early diagnosis of PE in TKA.

The hypothesis was that measuring SpO_2_ with a pulse oximeter after TKA is an effective and convenient method for the early diagnosis of PE.

## Methods

This study consecutively examined 1645 [782 unilateral, 416 simultaneous, and 447 sequential (done in the same admission with the interval of 1–2 weeks)] patients who underwent TKA by a single surgeon between January 2015 and November 2019. This study was a retrospective cohort study approved by the Institutional Review Board.

SpO_2_ was measured with a pulse oximeter in all patients until the postoperative day 2 (POD2). Postoperative SpO_2_ was measured using a pulse oximeter (Nonin Onyx Vantage 9590). NexGen Legacy Posterior Stabilized Flex Fixed Bearing (LPS Flex Fixed, Zimmer Warsaw, IN, USA) was used, and the mini-midvastus approach with minimally invasive surgery using quad-sparing™ instrumentation was utilized to perform TKA. We performed simultaneous TKA if a patient did not have comorbidities (such as coronary artery disease, chronic heart failure, chronic renal failure or stroke, etc.) or was under the age of 75 years. On the other hand, if a patient had comorbidities or was over the age of 75 years, we performed sequential TKA, and also sequential TKA was performed in the same admission with intervals of 1 or 2 weeks. Patients were hospitalized for up to 2 weeks after surgery for postoperative care, and postoperative rehabilitation begins on POD1 or 2 with a continuous passive motion (CPM) device or self-range of motion (ROM) exercise.

Pulse oximetry monitoring was started immediately when a patient returned to the ward with the 24-h monitoring device and then stopped if SpO_2_ was maintained at ≥ 95% without presenting symptoms of PE. On the other hand, regardless of the presenting symptoms, CTPA was performed for specific indications, including persistently low SpO_2_ < 95% (group 1), sudden decrease in SpO2 (group 2), and decrease in SpO_2_ after POD3 with presenting symptoms (group 3). Patients who received CTPA were categorized into two groups: PE diagnosed (group A) and non-diagnosed (group B) (Table 1) with a radiographic reading and confirmation of CTPA result from the department of radiology and specific laboratory studies.

**Table 1:** Patient demographics and clinical data

Variable	Group A (*n* = 20) PE	Group B (*n* = 38) No PE	*p* value
Age (years)	73.85 ± 6.78	73.87 ± 6.66	0.992
Gender: male/female	2/18	5/33	0.726
Body mass index (kg/cm^2^)	27.85 ± 3.64	27.14 ± 4.23	0.508
Operation site (Unilateral/Bilateral)	14/6	26/12	0.902
Comorbidities (*n*)			
Hypertension	17	32	0.937
Diabetes	6	11	0.933
Ischemic heart disease	1	1	0.638
Old CVA	0	3	0.197
Postoperative saturation (%)	85.0 ± 5.0	85.6 ± 5.8	0.696
Time to CTPA after surgery (days)	3.0 ± 2.15	2.63 ± 2.61	0.591
Duration of hospitalization (days)	16.75 ± 3.73	17.53 ± 5.48	0.527
Clinical symptoms (yes/no)	4/16	6/32	0.041

Data are presented as means with standard deviation (*p* < 0.05)

*CVA* cerebrovascular accident, *CTPA* computed tomographic pulmonary angiography, *PE* pulmonary embolism

Routine protocol was that a fondaparinux injection dose of 2.5 mg with subcutaneous injection from POD3 to POD7 to prevent venous thromboembolism was administered to all patients.

### Statistical analysis

Pearson’s chi-squared test was used to analyze the nominal variables. The independent *t*-test was used to compare continuous variables and determine statistical significance, the chi-squared test was performed for categorical variables, and weighted kappa coefficients were calculated. SPSS was used for statistical analysis, and the results were statistically significant at a *p* < 0.05.

## Results

Of the 1645 patients who underwent TKA, there were 20 patients with PE (1.2%) and symptomatic PE was observed in only 4 patients (0.24%). CTPA was performed in 58 (3.5%) patients, of whom 20 (34.5%) were diagnosed with PE (Table 1). Of the patients who received CTPA, no significant difference was found between group A (PE diagnosed) and group B (non-diagnosed) regarding age, sex, body mass index (BMI), surgery site, comorbidities (hypertension, diabetes, ischemic heart disease, and old cerebrovascular disease), the timing of CTPA, and clinical manifestations (Table 1).

In groups 1, 2, and 3, comprising 34, 21, and 3 patients, respectively, CTPA was performed 2.4, 2.6, and 8.3 days after TKA on an average, and 12 (34.3%), 8 (38.1%), and 0 patients were diagnosed with PE, respectively (Table 2).

**Table 2:** CTPA was performed in 58 patients (3.5%) out of 1646 patients

	Diagnosed with PE	CTPA performed per day
Group 1 (34 cases)	12 cases (34.3%)	2.4 day
Group 2 (21 cases)	8 cases (38.1%)	2.6 day
Group 3 (3 cases)	0 case (0%)	8.3 day

Group 1: persistent decreased SpO_2_ group (< 95%). Group 2: suddenly decreased SpO_2_. Group 3: decreased SpO_2_ with symptoms after POD3 days

*CTPA* computed tomographic pulmonary angiography, *PE* pulmonary embolism

*p* = 0.425

Of the 782 patients who underwent unilateral TKA, 38 received CTPA and 13 (34.2%) were diagnosed with PE. Of the 416 patients who underwent simultaneous TKA, 18 received CTPA and 6 (33.3%) were diagnosed with PE. Of the 447 patients who underwent sequential TKA, two received CTPA and 1 (50%) was diagnosed with PE. Of the patients diagnosed with PE, SpO_2_ was decreased without presenting symptoms in 16 patients (11 from group 1 and 5 from group 2). And there were four patient presenting with symptoms such as mild dyspnea and chest discomfort (one from group 1 and three from group 2) (Table 3). In this study, segmental PE was most frequently observed; there were 11 cases of segmental PE, 7 of subsegmental PE, and 2 of lobar PE.

**Table 3:** Twenty cases of pulmonary embolism

	Decreased SpO_2_ without symptoms	Decreased SpO_2_ with symptoms (mild chest discomfort, dyspnea)
Group 1 (12 patients)	11	1
Group 2 (8 patients)	5	3

Location of embolism: segmental artery (11 cases), subsegmental artery (7 cases), lobar artery (2 cases)

*p* = 0.255

**Fig. 1:**
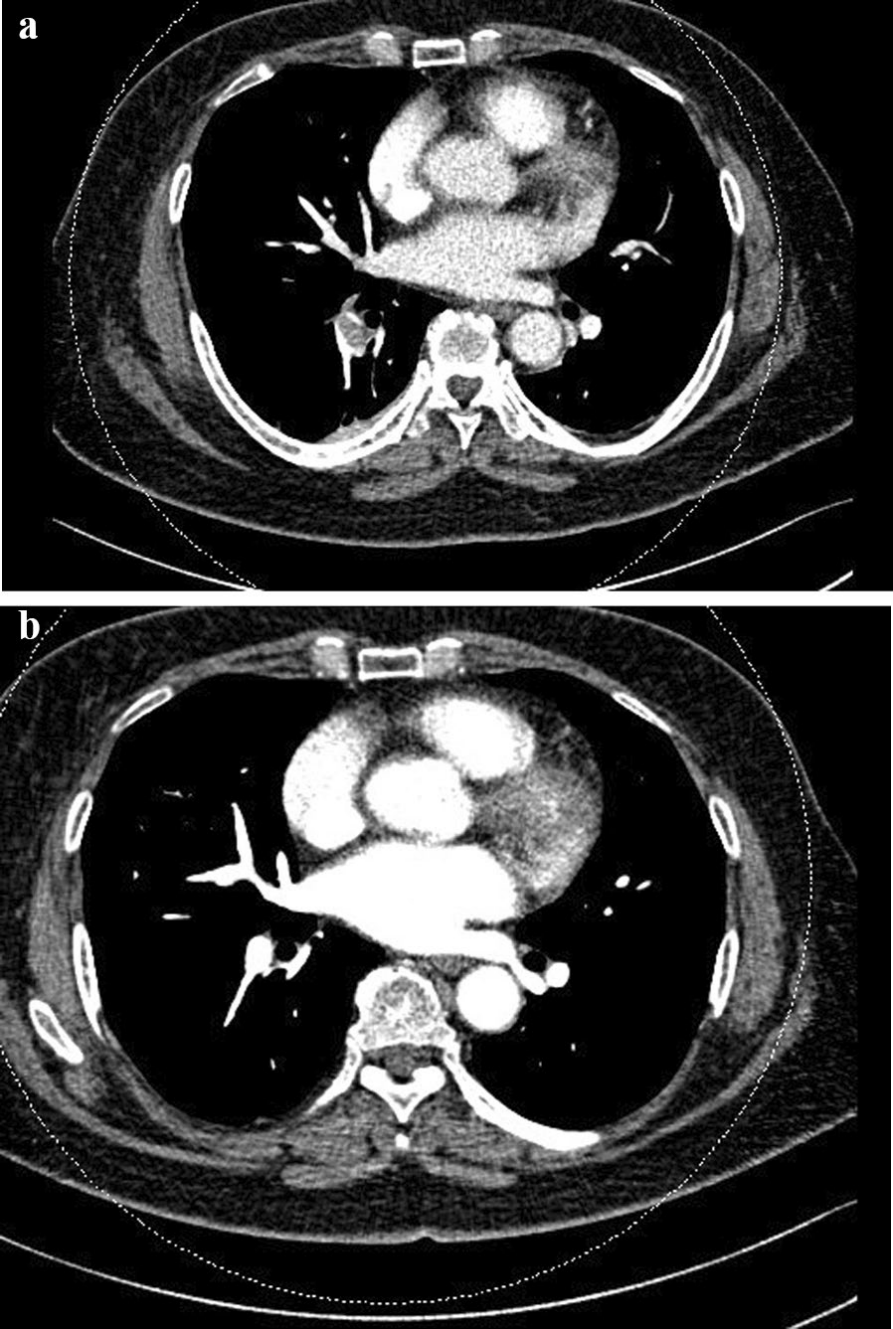
**a** 66-year-old female patient complained of dyspnea and showed SpO_2_ 90% on POD2 after both TKA. Pulmonary thromboembolism (PTE) in right lower lobe lobar artery was detected. **b** LMWH was administrated for 5 days followed by Pradaxa for 15 weeks. Pulmonary embolism disappeared after medication

As for the management of PE, it is important to decide first-line drug and when to start using the drug. Because it must be decided in consideration of various conditions, such as the patient’s underlying disease, it is important to have close consultation with internal medicine department. Four patients received unfractionated heparin (UFH) for 2–5 days (3 days on an average) followed by non-vitamin K antagonist oral anticoagulant (NOAC) for 1 day or until 32 weeks (27.3 weeks) (Table 4). Six patients received fondaparinux [low molecular weight heparin (LMWH)] for 2–10 days (5 days on average) followed by NOAC for 2–72 weeks (29.5 weeks). One patient received only LMWH for 13 days, and nine received only NOAC for 16–66 weeks (29.6 weeks) (Fig. 1). Medical treatment was stopped in one patient who received UFH for 3 days, followed by NOAC for 1 day due to severe hemorrhage and edema of the left leg. This patient underwent arthrocentesis and elastic band fixation; moreover, aspirin was administered 3 weeks after surgery when the patient was discharged without signs of bleeding. All patients were observed for at least 6 months after treatment. No case of morbidity or mortality was observed.

**Table 4:** Management of pulmonary embolism

	Number of patients	Average duration of treatment
Unfractionated heparin→non-vitamin K antagonist oral anticoagulant (NOAC)	4 patients	27.3 weeks
Unfractionated heparin→non-vitamin K antagonist oral anticoagulant (NOAC)	6 patients	29.5 weeks
Only low molecular weight heparin (LMWH)	1 patient	13 days
Only non-vitamin K antagonist oral anticoagulant (NOAC)	9 patients	29.6 weeks

## Discussion

Measuring SpO_2_ with a pulse oximeter after TKA is an effective and convenient method for the early diagnosis of PE. In this study, there were 20 patients with PE (1.2%) and symptomatic PE in only 4 patients (0.24%) of the 1645 patients who underwent TKA. Early diagnosis and management of PE are crucial as PE after TKA can cause serious complications including death.

Due to nonspecific and unreliable symptoms, signs, and laboratory evidence, an early diagnosis of PE remains difficult [12–14]. Failure of early diagnosis may lead to more severe clinical features, such as hemodynamic instability, right ventricular dysfunction, hypotension, obstructive shock, and obstructive shock, eventually causing death [15, 16]. Thus, early diagnosis and management of asymptomatic and mild PE can significantly reduce serious postoperative complications including death.

According to Watanabe et al., measuring postoperative d-dimer and cross-linked fibrin degradation products by leukocyte elastase (e-XDP) is helpful in predicting and diagnosing early postoperative VTE and PE [17]. d-dimer is the degradation product of cross-linked fibrin blood clots that increase in plasma when both coagulation and fibrinolysis are activated due to acute thrombosis [18]. e-XDP is a fibrin degradation product that is degraded by leukocyte elastase secreted by activated leukocytes and is useful in diagnosing disseminated intravascular coagulation and DVT [19, 20]. Watanabe et al. explained that the increase in POD1 e-XDP and POD4 d-dimer could be used for the early diagnosis of symptomatic VTE in patients with DVT after undergoing TKA. However, d-dimer levels can also be increased by any recent major surgery, hemorrhage, trauma, malignancy, and sepsis; thus, d-dimer levels cannot be used as a stand-alone diagnostic test for VTE [21]. On the other hand, the negative predictive values of d-dimer and e-XDP are above 98%; therefore, they are still useful as screening tests to exclude VTE. Thus, given that d-dimer and e-XDP are within normal plasma levels, no additional diagnosis examination is required [18–21]. Uchiyama et al. [22]. reported that dielectric blood coagulometry could help predict the occurrence of VTE after TKA. A platelet activation-dependent antigen, P-selectin, present on the platelet surface when platelets are activated by venous thrombosis, could also be an early marker for VTE [23].

When PE occurs, thrombosis in the pulmonary vasculature and outflow tract causes impaired blood flow and increased pulmonary artery pressure. This induces the secretion of humoral mediators, such as serotonin and thromboxane A2, from the platelets into the thrombus leading to vasoconstriction and an increase in pulmonary vascular resistance. Eventually, a V/Q mismatch causes hypoxemia [13, 24–26]. The presence of postoperative hypoxemia indicates the possible occurrence of PE; thus, an appropriate evaluation of hypoxemia may help diagnose early PE. SpO_2_ measured by a pulse oximeter allows for the non-invasive measurement of arterial oxygen saturation (SaO_2_), which helps evaluate hypoxemia in various circumstances [27]. In 2004, a study suggested eight PE rule-out criteria, including SpO_2_ > 94%, to reduce the excessive d-dimer measurements as the screening test for diagnosing possible PE [28]. In addition, SpO_2_ is included in the 11 evaluation criteria of the Pulmonary Embolism Severity Index suggested by Aujesky et al. [29].

A discrepancy between SpO_2_ measured using a pulse oximeter and actual SaO_2_ is inevitable. According to Wilson et al. [30], SpO_2_–SaO_2_ is 2.75% ± 3.1%; thus, the measured value of SpO_2_ is higher than that of SaO_2_. A total of 50% of the patients with 90–93% SpO_2_ had hypoxemia (SaO_2_ ≤ 90%); thus, using a pulse oximeter may lead to an underestimated diagnosis of hypoxemia [26, 30]. Therefore, a criterion for appropriately evaluating hypoxemia is required for a pulse oximeter. According to the retrospective evaluation of patients in the intensive care unit [14, 30], the cutoff value of SpO_2_ to detect hypoxemia (SaO_2_ ≤ 90%) was 94%. And the in-hospital morbidity rate due to PE was significantly reduced when SpO_2_ was ≥ 95% [23]. In addition, 95% SpO_2_, measured by a pulse oximeter, is the sole criterion to classify patients with PE into high- and low-risk groups [27, 29, 30]. Measurement errors may occur depending on the type of pulse oximeter; however, regardless of the measuring device, persistent SpO_2_ in the range of 95–100% confirms that a patient is not hypoxic [30].

Therefore, SpO2 ≥ 95% was used in this study to confirm that the patient did not have hypoxemia.A total of 58% of patients with SpO_2_ < 95% received CTPA, considering the possibility of hypoxemia due to PE. Of these patients, 20 (34.5%) were diagnosed early with PE. Of the 20 patients diagnosed with PE, 16 showed no symptoms and only 4 had mild dyspnea and chest discomfort, which are not typical clinical features of PE. Thus, utilization of SpO_2_ is clinically meaningful as a screening test for PE in patients who are either asymptomatic or present with mild symptoms.

In this study, if a patient takes medications such as anticoagulants or antiplatelet drugs, we stopped the drug some days before with delicate collaboration with internal medicine or neurology departments. After that LMWH (fondaparinux) was administered to all patients in POD3 to POD7 to prevent VTE. Either UFH followed by NOAC or NOAC alone was administered to patients diagnosed with PE before POD3. When PE was diagnosed during the administration of LMWH, LMWH was continued for some time before changing to NOAC. As for the management of PE, four patients received UFH for 2–5 days (3 days on average) followed by NOAC for 1 day to 32 weeks (27.3 weeks), six patients received LMWH (fondaparinux) for 2–10 days (5 days on an average) followed by NOAC for 2–72 weeks (29.5 weeks), one patient received only LMWH for 13 days, and nine patients received only NOAC for 16–66 weeks (29.6 weeks). Medical treatment was stopped in a patient who received UFH for 3 days, followed by NOAC for 1 day, due to severe hemorrhage and edema of the left leg. This patient underwent arthrocentesis and elastic band fixation; moreover, aspirin was administered 3 weeks after surgery when the patient was discharged without signs of bleeding.

Even though PE may also be asymptomatic and need any other treatment, some PE may become symptomatic and even cause death. Dentali et al. reported on their meta-analysis study; overall weighted mean prevalence of incidental PE was 2.6%, and the prevalence rose in high-risk patients who had cancer, DVT, and so on. In addition, results of some study showed high mortality rate of asymptomatic PE. Anja Boc et al. reported a study of the relationship between deep vein thrombosis and asymptomatic PE. In their study, about 36.1% of patients with DVT were related with asymptomatic PE and asymptomatic PE was one of the risk factors of recurrent PE. Trozan et al. reported 40% of recurrent PE was fatal. So, we need to check the PE with a non-invasive modality for detecting PE, if possible. This study shows we can detect PE early using non-invasive modalities such as pulse oximetry.

There are some limitations to this study. First, the limitation of this study is that the study period (2015–2019) is relatively short, and there are characteristics of the disease that require long-term follow-up, and so it has the disadvantage that it does not reflect the overall natural history of the disease. Also, there is a limitation that other causes that may affect the PE and condition, such as underlying comorbidities, deep vein thrombosis and methods of prophlactics, opioid use, or age etc., could not be observed or analyzed. We did not check the Doppler sonography for detecting DVT. Also we did not check all patients’ SaO_2_, and it might underestimate some patients according to Wilson et al.

This study’s conclusions were drawn given that it is a sample only of a single surgeon’s patients with TKA.

Patients diagnosed with PE can easily be overlooked due to the presentation of atypical symptoms.

## Conclusions

Measuring SpO_2_ with a pulse oximeter is clinically meaningful to determine PE after TKA. In addition, there was no morbidity or mortality case after early diagnosis with early stage CTPA and management of PE. In conclusion, measuring SpO_2_ with a pulse oximeter after TKA is an effective and convenient method for the early diagnosis of PE.

## Data Availability

The datasets used and/or analyzed during the current study are available from the corresponding author on reasonable request.

## References

[1] Singh JA, Shaohua Y, Chen L, Cleveland JD (2019). Rates of total joint replacement in the United States: future projections to 2020–2040 using the national inpatient sample. J Rheumatol.

[2] Geerts WH, Bergqvist D, Pineo GF, Heit JA, Samama CM, Lassen MR (2008). Prevention of venous thromboembolism: American College of Chest Physicians evidence-based clinical practice guidelines (8 Edition). Chest.

[3] Haake DA, Berkman SA (1989). Venous thromboembolic disease after hip surgery: risk factors, prophylaxis, and diagnosis. Clin Orthop Relat Res.

[4] SooHoo NF, Lieberman JR, Ko CY, Zingmond DS (2006). Factors predicting complication rates following total knee replacement. J Bone Joint Surg Am.

[5] Cote MP, Chen A, Jiang Y, Cheng V, Lieberman JR (2017). Persistent pulmonary embolism rates following total knee arthroplasty even with prophylactic anticoagulants. J Arthroplasty.

[6] Stein PD, Athanasoulis C, Alavi A, Greenspan RH, Hales CA, Saltzman HA (1992). Complications and validity of pulmonary angiography in acute pulmonary embolism. Circulation.

[7] Hirsh J, Hoak J (1996). Management of deep vein thrombosis and pulmonary embolism: a statement for healthcare professionals. Council on Thrombosis (in consultation with the Council on Cardiovascular Radiology), American Heart Association. Circulation.

[8] Goldhaber SZ, Visani L, De Rosa M (1999). Acute pulmonary embolism: clinical outcomes in the International Cooperative Pulmonary Embolism Registry (ICOPER). Lancet.

[9] An VVG, Phan K, Levy YD, Bruce WJM (2016). Aspirin as thromboprophylaxis in hip and knee arthroplasty: a systematic review and meta-analysis. J Arthroplasty.

[10] Cusick LA, Beverland DE (2009). The incidence of fatal pulmonary embolism after primary hip and knee replacement in a consecutive series of 4253 patients. J Bone Joint Surg Br.

[11] Zahir U, Sterling RS, Pellegrini VD, Forte ML (2013). Inpatient pulmonary embolism after elective primary total hip and knee arthroplasty in the United States. J Bone Joint Surg Am.

[12] Barco S, Ende-Verhaar YM, Becattini C, Jimenez D, Lankeit M, Huisman MV (2018). Differential impact of syncope on the prognosis of patients with acute pulmonary embolism: a systematic review and meta-analysis. Eur Heart J.

[13] Desciak MC, Martin DE (2011). Perioperative pulmonary embolism: diagnosis and anesthetic management. J Clin Anesth.

[14] Dentali F, Ageno W, Becattini C, Galli L, Gianni M, Riva N (2010). Prevalence and clinical history of incidental, asymptomatic pulmonary embolism: a meta-analysis. Thromb Res.

[15] Pollack CV, Schreiber D, Goldhaber SZ, Slattery D, Fanikos J, O'Neil BJ (2011). Clinical characteristics, management, and outcomes of patients diagnosed with acute pulmonary embolism in the emergency department: initial report of EMPEROR (Multicenter Emergency Medicine Pulmonary Embolism in the Real World Registry). J Am Coll Cardiol.

[16] Laack TA, Goyal DG (2004). Pulmonary embolism: an unsuspected killer. Emerg Med Clin North Am.

[17] Watanabe H, Madoiwa S, Sekiya H, Nagahama Y, Matsuura S, Kariya Y (2011). Predictive blood coagulation markers for early diagnosis of venous thromboembolism after total knee joint replacement. Thromb Res.

[18] Perrier A, Roy P, Aujesky D, Chagnon I, Howarth N, Gourdier A (2004). Diagnosing pulmonary embolism in outpatients with clinical assessment, D-dimer measurement, venous ultrasound, and helical computed tomography: a multicenter management study. Am J Med.

[19] Matsumoto T, Wada H, Nobori T, Nakatani K, Onishi K, Nishikawa M (2005). Elevated plasma levels of fibrin degradation products by granulocyte-derived elastase in patients with disseminated intravascular coagulation. Clin Appl Thromb Hemost.

[20] Madoiwa S, Tanaka H, Nagahama Y, Dokai M, Kashiwakura Y, Ishiwata A (2011). Degradation of cross-linked fibrin by leukocyte elastase as alternative pathway for plasmin-mediated fibrinolysis in sepsis-induced disseminated intravascular coagulation. Thromb Res.

[21] Stein PD, Hull RD, Patel KC, Olson RE, Ghali WA, Brant R (2004). D-dimer for the exclusion of acute venous thrombosis and pulmonary embolism: a systematic review. Ann Intern Med.

[22] Uchiyama H, Inoue Y, Uchimura I, Nakamura T, Kudo T, Muneta T (2017). Prediction of venous thromboembolism after total knee arthroplasty using dielectric blood coagulometry. Ann Vasc Surg.

[23] Yang L, Wang C, Lee T, Lin F, Yang B, Lin C (2002). Early diagnosis of deep vein thrombosis in female patients who undergo total knee arthroplasty with measurement of P-selectin activation. J Vasc Surg.

[24] Burrowes KS, Clark AR, Tawhai MH (2011). Blood flow redistribution and ventilation-perfusion mismatch during embolic pulmonary arterial occlusion. Pulm Circ.

[25] Smulders YM (2000). Pathophysiology and treatment of haemodynamic instability in acute pulmonary embolism: the pivotal role of pulmonary vasoconstriction. Cardiovasc Res.

[26] Lankhaar J, Westerhof N, Faes TJC, Marques KMJ, Marcus JT, Postmus PE (2006). Quantification of right ventricular afterload in patients with and without pulmonary hypertension. Am J Physiol Heart Circ Physiol.

[27] Van de Louw A, Cracco C, Cerf C, Harf A, Duvaldestin P, Lemaire F (2001). Accuracy of pulse oximetry in the intensive care unit. Intensive Care Med.

[28] Kline JA, Mitchell AM, Kabrhel C, Richman PB, Courtney DM (2004). Clinical criteria to prevent unnecessary diagnostic testing in emergency department patients with suspected pulmonary embolism. J Thromb Haemost.

[29] Aujesky D, Perrier A, Roy P-, Stone RA, Cornuz J, Meyer G (2007). Validation of a clinical prognostic model to identify low-risk patients with pulmonary embolism. J Intern Med.

[30] Konstantinides SV, Meyer G, Becattini C, Bueno H, Geersing G, Harjola V (2020). 2019 ESC Guidelines for the diagnosis and management of acute pulmonary embolism developed in collaboration with the European Respiratory Society (ERS). Eur Heart J.

